# Some Observations on the Use of Colchicum, Tending to Prove That Gout Can Be Cured by Smaller Doses of That Medicine Than Are Exhibited by Those Who Not Only Reprobate the Use of What They Prescribe, but Deny It Any "Specific Virtue:" with Some Cursory Objections to the Gastro-Hepatic Pathology

**Published:** 1821-11

**Authors:** R. W. Bampfield

**Affiliations:** one of the Surgeons to the Royal Metropolitan Infirmary for Sick Children, &c. &c.


THE LONDON

### Medical and Physical Journal.

### Original Communications, Select Observations, etc.

---

*Some Observations on the Use of Colchicum, tending to prove that Gout can be cured by smaller Doses of that Medicine than are exhibited by those who not only reprobate the Use of what they prescribe, but deny it any "specific Virtue:" with some cursory Objections to the Gastro-hepatic Pathology.* By R. W. BAMPFIELD, Esq. one of the Surgeons to the Royal Metropolitan Infirmary for Sick Children, &c. &c.

SEVERAL years have elapsed since the property of colchicum to speedily cure the gouty paroxysm became known, principally through the medium of the Medical and Physical Journal, and the empirics proclaimed to the world, what is indeed truth, that "medicine cannot boast of a much more important discovery;" yet, although its efficacy and power have been clearly established by the evidence of as many instances of its unequivocal effects as ever supported a medical fact, this concurrence has not produced such an uniformity of opinion and practice as the evidence seems to sanction and demand; nor has it led to the general adoption of this medicine in the treatment of gout. In the mean time, numerous nostrum venders, availing themselves of the publicity of the discovery, have taken advantage of the supineness and discrepancies of professional men, to enrich themselves by selling this "specific for the cure of gout," under the titles of "Reynold's Specific," "Wilson's Tincture," "Light's Royal Specific," "W—'s Medicine for the Gout," "Perry's Specific," &c. &c.; and, while some practitioners have been slow to recommend, or prompt in objecting to the administration of colchicum, many patients have terminated their acute sufferings by having recourse to its use in the shape of a nostrum, and have become advocates, irresistibly eloquent, in pleading the cause of selfish individuals. It appears to me that this order of things should not exist without an examination into the nature of its causes, and the force of the objections to the use of colchicum as a remedy for gout. The following have occurred to me as among the principal causes that influence the conduct of the profession:—

Prejudice, or previous belief; scepticism; some fatal and unexpected results from large doses of the medicine; deference to the authority of great names.

Prejudice is two-fold: it may sway the mind of the patient or of his doctor. On the part of the patient it may be excused, if, from experience of the inefficacy of medicine, he has concluded gout to be the *opprobrium medicorum*, or, at all events, that no remedy is a jot superior to patience and flannel; or if, when not interrupted in its natural course, it has subsequently bestowed immunity from all other diseases, he may have a strong previous belief that such consequences would not follow a hasty cure of the paroxysm. In whatever manner this prejudice arises, if it cannot be removed by reason, a relation of facts, and the confidence the patient usually places in his physician, we must leave him to the powerful argument of a severe fit of pain. Medical prejudice is inexcusable; for, as it implies firm belief without experiment, which it excludes, we dare not hope to remove it, except by the desertion of patients, and the consequent strong appeals of self-interest; unless the prejudiced become subject to gout, and are roused by a contemplation of the loss of locomotion, from a successive destruction of their joints.

Medical scepticism on the virtues of new remedies, is, to a certain extent, justified by experience; for few, who have practised a quarter of a century, but must have witnessed the ephemeral life of favourites of fashion; and all, who have read the history of pharmacy, must know how many medicines, wonder-working in their day, have been consigned to utter neglect and oblivion. Medical scepticism is also rational, and (like doubt) becomes the parent of truth, when it is accompanied with experimental investigation and patient inquiry: without them, it must be nearly allied to prejudice.

Fatal results have speedily followed the use of large doses (more especially of the sedimentous portion,) of vinum colchici, and we apprehend the two-drachm doses of its discoverers were incompatible with the patient's safety. I have twice witnessed death to ensue on the following day after a drachm had been administered over night, in two cases of dropsy; yet I have not seen such a consequence from its employment in gout. The objection to its use, which arises from its being a poison, should not apply with greater force against it than against any other deleterious medicine. Opium will poison in large doses, yet every body prescribes it. The objection can only be urged against unsafe doses; and one of the objects of this paper is to point out the successful results of small doses of vinum colchici, as cannot alarm the most timid, in carrying off the regular paroxysm of gout; a circumstance which does not appear to be generally known.

The authority of great names is, indeed, diminishing in this age of strict inquiry: on the present subject, the public possess the authority of Dr. Scudamore for entertaining a belief, that "the effects of colchicum in gout are altogether unsatisfactory,"* whilst, in my own practice, it has uniformly proved most satisfactory: it will, therefore, be another object to demonstrate that Dr. S. administers as much colchicum as any other practitioner, and to establish a conviction that he cures gout by the same remedy he has condemned as "altogether unsatisfactory." In the formula† for the diuretic purgative draught, at page 186 of Dr. S.'s scientific Treatise on Gout, he prescribes from four to twelve drachms of the acetum colchici, to be taken in divided doses in twenty-four hours, of which the medium quantity would be one ounce a-day. Of this form of exhibition it is observed, at p. 200, "From a very careful investigation into the properties of colchicum, I am led to think that *the acetic acid takes up all its active properties, and that given in the formula which I have already mentioned, it produces all the good effects of which the medicine in its other forms is capable,* and is not chargeable with any one ill consequence." In the formula alluded to, "the acetic acid is neutralized by magnesia, and the colchicum, brought to the state of a mere solution in water, by having its acid menstruum neutralized, *is in the most favourable state of preparation in which it can possibly be administered.*" (Note, p. 186.) Notwithstanding this free use of the colchicum "in its most favourable state," and the great success of the treatment of which it forms a part, Dr. S., in speaking more particularly of this medicine, given "in free doses," (the exact quantity is not mentioned,) says, its "effects were altogether unsatisfactory;" and he "observed that the stomach was irritated; an increased fur of the tongue, with thirst, were produced; and no certain action of the bowels occurred." (P. 193.) In my practice, such effects have not followed; nor have I had occasion "to charge" the vin. colchici

* Scudamore *on Gout*, p. 193.

† "I have experienced the most remarkable success from a draught of magn. gr. xv. ad xx.; magn. sulphat. ʒj. ad ʒij.; acet. colch. ʒj. ad ʒij.; with any distilled water the most agreeable, and sweetened with syrup or extr. glycyrrh. It should be repeated at intervals of four, six, or eight, hours."—P. 186, *op. cit.*

"with any one ill consequence;" and it appears to me, that the acetum prescribed by the Doctor is chiefly entitled to the credit of carrying off the gouty pain and inflammation; for who has experienced gout to be certainly and speedily cured by magn. sulph., magnesia, and an "agreeable distilled water?" and, if constipation be guarded against in such cases as in those in which the vin. colchici is exhibited, the furred tongue, irritated stomach, and thirst, will not occur: besides, where the vin. colch. is used, purgatives are not generally necessary.

I shall now proceed to demonstrate that Dr. S. administers larger doses of colchicum than the generality of practitioners who prefer the wine; and, in adducing the proofs, I will concede that vinegar and wine are equally good menstrua, and that they extract *all the active properties* of the bulb of colchicum that is macerated in them, notwithstanding the fluid menstruum in the vinous preparation scarcely covers the bulb, and the vinegar has so high a reputation for extracting its gouty virtues.

In making the acetum colchici, one ounce of the bulb is macerated in eight ounces of the vinegar, to which one ounce of spirit is afterwards added. *Pharm. Londin.* Therefore, if we calculate the proportion of colchicum to the quantity of fluid menstruum directed for extracting its medicinal properties, the result, by the rule of Three, is as follows, on Dr. S.'s half ounce, one, and one and a half ounce, quantities per day:

| | oz. Acet. Col. | | dr. Colchicum | | oz. | | gr. |
|---|---|---|---|---|---|---|---|
| If | 9 | : | 8 | :: | 1 | :: | $26\frac{6}{9}$, Dr. S.'s minimum quantity: |

*i. e.* If 9 oz. of acet. colch. contain "all the active properties" of 8 drachms of colchicum, how much will half an ounce contain? Answer,—$26\frac{6}{9}$ grains, and so on.

| | | | | | | | |
|---|---|---|---|---|---|---|---|
| If | 9 | : | 8 | :: | 1 | :: | $53\frac{1}{3}$, medium; |
| If | 9 | : | 8 | :: | $1\frac{1}{2}$ | :: | 80, maximum quantity in 24 |

hours, in divided doses.

In preparing the vin. colchici, the druggists,* from whom I have purchased it, have macerated one pound of the bulb in a pint of sherry wine; and I have administered, most commonly, from 20 to 30 minims for three nights in succession, but I have in one case given 60 minims; which is the dose usually prescribed by Sir E. Home,† and which, in the generality of cases, is unnecessarily large, and may be unsafe.

| | oz. Vin. | | oz. Colch. | | m. | | gr. |
|---|---|---|---|---|---|---|---|
| If | 16 | : | 16 | :: | 20 | :: | 20, the minimum dose, |
| If | 16 | : | 16 | :: | 40 | :: | 40, medium ditto, |
| If | 16 | : | 16 | :: | 60 | :: | 60, maximum ditto, and |

quantity in 24 hours.

* Godfrey and Cooke.

† See the *London Medical Repository.*

Hence, in the relative quantities given by Dr. S. and myself, in twenty-four hours, his exceeds mine by exactly one-fourth, or twenty-five per cent.; as, in one ounce of acet. colchici, he administers "all the active properties" of 53⅓ grains of colchicum, while, in the medium dose of 40 minims, I only administer the active properties of 40 grains; and, should the formula* in Dr. Paris's Pharmacology for making the vin. colchici be received, as probably it will, Dr. Scudamore will then use much more than double, or 160 per cent. more colchicum than would be administered by those who use the wine.

One objection must be noticed, which I have heard urged both by patients and practitioners: it is, that, when the paroxysm is carried off by the vin. colch. directly on its invasion, the constitution does not finally feel so vigorous after it, as when it has recovered from a prolonged attack. With such patients, the objection has been obviated to their satisfaction, by allowing the gouty paroxysm to continue a few days before the medicine is prescribed. After Mr. R. had used the colchicum five years, he formed this opinion relative to his attack in January of this year, and, when the paroxysm recurred in May, he suffered it to take its uninterrupted course five days, and then removed it by three successive doses of thirty minims of the wine. The result was, that his health became restored as perfectly as after any former attack.

Two great benefits arising from the speedy cure of the gouty paroxysm, may be boldly claimed, and adduced for the consideration of the opponents of this method of cure: the first is, that it scarcely allows any time for the chance of such a dangerous, and often fatal, occurrence as retrocedent gout, with which the protracted duration of the disease menaces the patient at all periods of its course; as is evinced in the Hon. V. G.'s case. The second is, that it prevents the formation of chalk-stones. W. B. (Case 9,) had almost all the joints of his fingers disabled and distorted by their successive growth; but, since the treatment by vin. colch. was adopted in 1817, the disposition to form them has been removed: nor has this disposition appeared in any of my patients, who have been cured by the wine.

The gastro-hepatic pathology of gout has many able and learned advocates, yet general and particular objections may be reasonably urged against its correctness, even if Dr. S.'s definitions of the proximate cause of disease in general, and of gout in particular, be selected to regulate the discussion.—"The proximate cause of a disease," says he, "must not only be something which is *invariably antecedent*, but also that which is *distinctly peculiar* to such disease." P. 130. And he remarks,

* In this, half a pound of the colchicum is macerated in a pint of wine.

"We are now brought to the general conclusion, that gout is a disease depending upon a redundancy of blood with relation to the powers of the circulation, particularly affecting the system of the vena portarum, and the consequent functions of the liver, together with the production of a morbid change in the secreted products of the alimentary canal in general, and of the kidneys in particular." It may be observed, that gouty subjects generally possess strong digestive functions and athletic forms, whilst the habitual dyspeptics are seldom subject to gout. Mr. Lewis, an old martyr to gout, and Mr. Short, his surgeon, write me from Warwickshire, that I may look in vain to deranged digestive functions for the cause of his long and frequent attacks of gout. The morbid change of the secreted products of the alimentary canal and kidneys, and derangement of the hepatic functions, are not "distinctly peculiar" to gout, but occur in all diseases of the class Pyrexia. Nor are those visceral derangements of functions "invariably antecedent" to gout. A patient of mine fell from his horse, and on the same day became attacked with gout. Captain H—n is often invaded suddenly by gout after getting wet or enduring fatigue; and he assures me, *his appetite and digestion are more vigorous during the arthritic paroxysm than at any other time.* Mr. R. wore a tight boot, which irritated his great toe, and on the following night the arthritic invasion supervened. In these and such cases there was no "antecedence" of deranged chylopoïetic viscera. Besides, in the treatment of gout by the vin. colch. I have never prescribed any medicine to correct, regulate, or improve, the digestive functions, except to obviate constipation when present; yet, if they have been partially or wholly deranged by symptomatic fever, they have always recovered as soon as after any other febrile disease. W. Bunker (Case 9,) was emaciated and dyspeptic from being confined half the year to bed by gout; but, since the cure by colch. has been adopted, he has become stout and corpulent, and never complains of indigestion.

In the constitutional treatment of all the local diseases mentioned by Mr. Abernethy and others, we do not possess any other means of curing the local diseases except by such medicines as regulate and improve the digestive functions: in gout, on the contrary, we can speedily remove the local disease without paying any further attention to the digestive organs than to obviate constipation, which experience convinces us we could not effect, if the local disease depended upon, and were caused by, constitutional derangement of the digestive processes.

Although I have availed myself of leeches and purgatives in some of the following cases, there is no doubt left in my mind of the vin. colchici having been the principal agent in carrying off the gouty paroxysm.

*Cases of Gout.*

Case I.—Mr. Sandison, æt. 16, April 4th, 1818, has sustained a few attacks of gout; the last was carried off by Reynold's specific, but in the present invasion that medicine completely failed. The great toes, the feet, and the knees, are affected with pain, swelling, and redness; he feels considerable pain in the stomach and bowels, which are constipated; and the fever is severe, with anorexia.

℞. P. rhei et jalapæ, āā ℈ss.; Træ. rhei, ʒiij.; Aq. menth. pip. ℥j.; ft. haust. st. sum.

℞. Vin. colch. m. xx.; Mist. Camp. ʒx.; Spt. lav. co. ʒj.; Ft. haust. omni nocte sumendus. The diet to be low.

*5th.*—The gouty affections of the stomach and bowels have subsided; the fever has abated, as well as the pains in the joints; and, after repeating the colchicum on this and the following evening, the gouty paroxysm was entirely carried off.

Case II.—Miss Davis, æt. 19, Oct. 17th, 1818, appears to have inherited a predisposition to gout from her mother and her ancestors, and has sustained one painful attack about three years ago. The great toe of the right foot, the foot, and knee, became swelled and painful yesterday, and she is affected with slight constitutional fever and loss of appetite; bowels regular. She was sent three draughts, of Vin. colch. m. xx.; Aquæ, ℥iss.; Træ. card. co. ʒss.; and directed to take one every night, but she only took two, when the paroxysm was carried off.

Case III.—Mrs. Jackson, æt. 40, November 28th, 1818, has been much subject to the gout, and was invaded by the present attack twelve days ago: both the great toes and the right wrist are swelled and painful; the knees are stiff, and she is confined to bed; the bowels are costive, and she has a slight degree of fever, with anorexia and loss of sleep. Capit. haustus aperiens statim, et vin. colchici m. xx. in aquæ ℥jss. omni nocte. The gouty paroxysm, and its constitutional effects, gradually subsided; and, on the 2d of December, no symptoms but a stiffness of the wrist remained; and, on the 3d, she was quite well.

She had a recurrence of gout on the 2d of January, 1819, which was carried off by two doses of vin. colchici, m. xxv. each.

Case IV.—John Collins, æt. 60, June 12th, 1818, is affected with considerable pain and swelling of both great toes; the wrists are also affected; and the gouty paroxysm is attended with fever, and has been preceded by dyspepsia. ℞. Vin. colc. m. xx.; Aquæ, ℥jss. M. ft. haust. omni nocte sumendus.

*June 14th.*—The gouty pains have abated, but the fever is maintained, perhaps from the bowels being confined. Capit. haustus cathart. statim, et rep. vin. colchici, m. xx. On the 16th, the gouty paroxysm was carried off, and no further medicine was given.

Case V.—E. Jackson, æt. 61, has been long subject to an anomalous derangement of the digestive functions; and, on the 28th of May, 1818, was attacked with gout in the great toe of the right foot and in the right wrist, the pain of which was accompanied with fever and loss of appetite, and she was confined to bed; the bowels regular. ℞. Vin. colch. m. xx.; Aquæ, ℥jss.; ft. haustus omni nocte sumendus. The draught was repeated six nights before the pain was entirely removed.

Case VI.—Mr. D. Thomas, æt. 45, March 4th, 1818, has been suffering from gout and cough; for the cure of which he had been under a physician, who happened to alarm his friends by giving an opinion that the case would terminate in consumption: the gout now affects his knees and right hand, and the left ankle and foot are much inflamed and very painful; the cough is troublesome, and he has considerable fever. Appl. hirudines sex talo et pedi sinistro. Capit. haustus cath. mane. ℞. Vin. colch. m. xx.; Liq. ant. tart. m. xx.; Aquæ menth. pip. ℥jss.; ft. haust. h. s. omni nocte sumendus.

*March 5th.*—The leeches bled freely, the cathartic has operated, and he has perspired in the night; he is better to-day. Rep. haust. cum vini colchici, m. xx. &c.

*6th.*—The gouty affections are much relieved; he has very little fever; he sleeps better; the cough is moist, and less troublesome; bowels open. Rep. haustus.

*7th.*—Continues to amend. Rep. haustus.

*8th.*—The gouty paroxysm and pain are completely carried off, as well as the fever; the joints are rather stiff, and the cough is troublesome, particularly at night. The joints were fomented and rubbed with flannel; some squills and ipecacuanha were given every six hours, and a pill of Pil. sapon. cū. opio, gr. ijss. omni nocte; and in three weeks the cough was cured.

Case VII.—J. Patterson, æt. 57, October 30th, 1817. This patient had lost one leg, but the great toe of the other was affected by gout, as well as his hands and fingers, in the joints of many of which concretions had formed, from previous attacks; he had been ill several days. He took vin. colchici, m. xx. in water, for three nights, and the paroxysm was carried off.

Case VIII.—Mrs. Farlow, æt. 71, August 5th, 1817. Has had the gout about twenty-five years, and is now afflicted with severe gouty pain and swelling of the right arm, especially of the wrist, and in both feet, so that she cannot walk; she has not any particular degree of fever, but feels nervous and unwell generally. ℞. Vin. colchici, m. xxx.; Aquæ, ℥jss.; ft. haustus, h. s. s.

*August 6th.*—The medicine appears to have acted twice on the bowels, and has procured so much relief, that I found her sitting up. Rep. haustus.

*7th.*—Is much easier, and the swellings subside. Rep. haustus. On the 8th, the gouty pain was quite removed, but the swelling of the right wrist had not wholly subsided. The draught was omitted, but friction was used to the wrist.

This patient had another attack of gout on the 6th of April, 1818, which was carried off by three doses of twenty minims of vin. colchici.

CASE IX.—W. Bunker, æt. 47, a stout athletic man, has been subject to gout twenty-four years, and concretions have formed on several of the joints of the fingers. Since he became my patient, he has had two severe attacks of gout every year, which have been carried off by twenty-five to sixty minims of vinum colchici, taken on three or four successive nights.

*Nov. 23d,* 1818.—I visited him in company with the late Mr. Gatcombe: the knees, the right hand and wrist, were much inflamed, swelled, and painful; he had considerable fever, attended with thirst, loss of appetite, and sleep. Capt. h. carth. statim, et vin. colchici, ʒss. in aqua, hora somni.

*24th.*—The knees, right hand, and wrist, are less inflamed and painful; the right elbow is becoming swelled and painful; the bowels are open; the urine is scanty, although, he says, "he voids a gallon a-night when he is well;" the fever has abated. Rep. haustus c. vin. colchici.

*25th* and *26th.*—The gouty inflammation, pain, and fever, have much abated, and he can now sit up. He continued the draught until the 28th, when the paroxysm was carried off, and he recovered without any further medicine. In one only of the subsequent attacks, I have increased the quantity of vin. colchici to sixty minims.

CASE X.—Mr. H. Fisher, æt. 58, of a stout full habit, on May 2d, 1818, was confined to bed with a gouty inflammation of the right great toe, to which he was subject, with some fever, loss of appetite, and sleep; his bowels were regular. ℞. Vin. colchici, m. xxv.; Aquæ, ℥jss. M. ft. haustus omni nocte sumendus. He continued it three nights, and the paroxysm disappeared.

CASE XI.—The Hon. V. G. had been suffering from gout about seven weeks, in his joints, and at one period it invaded the stomach. On the 12th December, 1818, I was sent to him, and found the ankle, foot, and toes, of one extremity, and the knee of the other, considerably swelled, inflamed, and painful: the redness was greatest about the ankle. He told me that, among other remedies, he had applied thirty-six leeches, and he was now taking infusion and tincture of hops. ℞. Vin. colchici, ʒss.; Aq. menth. viridis, ℥jss.; Spt. lav. co. ʒss. M. ft. haustus omni nocte sumendus.

*Dec.* 13*th.*—He was rather better, and the redness had moved to the upper part of the great toe, which was more tender than yesterday. Rep. haustus, et utatur frictio pedi, talo et genu.

14*th.*—Is much easier; the swelling subsides, and the redness of the toe is fainter.—Rep. haustus.

15*th.*—The pain has entirely left him; the swelling subsides; and he, this morning, put his slipper up at the heel, and, taking three draughts with him, set out on a journey of seventy miles. The swelling and stiffness gradually disappeared, and he took the other three draughts.

I have met with some instances of a combined form of gout with rheumatism, or of rheumatism in a gouty habit, which would not yield to the colchicum in the short periods the rest have done; and with some, where it did not effect a cure at all. Mrs. M'Gregor caught cold during the use of mercury, and an attack of rheumatic gout, in one of its most violent forms, ensued,—attended with fever and its consequences: the great toes, as well as all the joints and hands, were inflamed in succession; and, with the aid of leeches repeatedly applied, laxatives, and diaphoretics, the vin. colchici did not effect a cure in less than three weeks. In Mrs. D. Peake's mixed case, neither the wine of the seeds or bulb succeeded; nor have these medicines proved so efficacious in cases of pure rheumatism as I had been induced to expect.

It should be observed, that Dr. Scudamore's treatment and mine are similar in effect, as both cure the paroxysm of gout in a few days, and that I have been induced to attribute this effect to the same remedy; but the inference might appear to be drawn from a negative proof alone, as I have only denied that his draught will cure gout in a few days, if the acet. colchici be withdrawn from it. This negative proof may not be satisfactory, unless it be coupled with a positive one, that acet. colchici will cure gout without the other ingredients of his draught. To ascertain this, I gave Mrs. Jackson a draught of acet. colch. et aq. menth. pip. āā ʒvj. three nights in succession, and the paroxysm was carried off. In one of W. Bunker's attacks, (whose gouty habit has been noticed,) I gave him an ounce of each, and in four nights the paroxysm was carried off; but he complained of its having given him pains in the bowels, and that "it did not carry off gout so pleasantly as the other medicine,"—meaning the wine; nor would he take it again. I therefore desisted from further experiment.

Dr. Scudamore further denies that colchicum possesses either

"specific virtues or operation in the gout," (p. 193;) yet, if Dr. Parr's definition of a specific be admitted, both must be assigned to it, for, without doubt, "it is peculiarly adapted to curing the gouty paroxysm;" but no medical practitioner has been so absurd as to claim for the colchicum the property of curing a deranged liver or digestive functions, not even if they should ensue from an attack of gout.

*37, Bedford-street, Covent Garden; Sept. 14th,* 1821.

